# Cross-Classification of Human Urinary Lipidome by Sex, Age, and Body Mass Index

**DOI:** 10.1371/journal.pone.0168188

**Published:** 2016-12-14

**Authors:** Kazuo Okemoto, Keiko Maekawa, Yoko Tajima, Masahiro Tohkin, Yoshiro Saito

**Affiliations:** 1 Division of Medicinal Safety Science, National Institute of Health Sciences, Setagaya, Tokyo, Japan; 2 Graduate School of Pharmaceutical Sciences, Nagoya City University, Nagoya, Aichi, Japan; Georgetown University, UNITED STATES

## Abstract

Technological advancements in past decades have led to the development of integrative analytical approaches to lipidomics, such as liquid chromatography-mass spectrometry (LC/MS), and information about biogenic lipids is rapidly accumulating. Although several cohort-based studies have been conducted on the composition of urinary lipidome, the data on urinary lipids cross-classified by sex, age, and body mass index (BMI) are insufficient to screen for various abnormalities. To promote the development of urinary lipid metabolome-based diagnostic assay, we analyzed 60 urine samples from healthy white adults (young (c.a., 30 years) and old (c.a., 60 years) men/women) using LC/MS. Women had a higher urinary concentration of omega-3 12-lipoxygenase (LOX)-generated oxylipins with anti-inflammatory activity compared to men. In addition, young women showed increased abundance of poly-unsaturated fatty acids (PUFAs) and cytochrome P450 (P450)-produced oxylipins with anti-hypertensive activity compared with young men, whereas elderly women exhibited higher concentration of 5-LOX-generated anti-inflammatory oxylipins than elderly men. There were no significant differences in urinary oxylipin levels between young and old subjects or between subjects with low and high BMI. Our findings suggest that sex, but neither ages nor BMI could be a confounding factor for measuring the composition of urinary lipid metabolites in the healthy population. The information showed contribute to the development of reliable biomarker findings from urine.

## Introduction

Metabolomics is a relatively recent research area focused on the systemic profiling of small-molecule metabolites. Lipidomics is a lipid-targeting subarea of metabolomics that involves comprehensive analyses of pathways and interactions of cellular lipids in biological systems. Recent technological developments in mass spectrometry (MS) and chromatography have considerably advanced the application of lipid metabolic profiling in complex biological samples [1]. Because the structure of lipid molecules, in contrast to that of genome-encoded proteins, cannot be studied via direct manipulation of the respective genes, lipidomics is now considered to be one of the most important and rapidly developing research areas.

Owing to their considerable structural diversity, lipids are important players in a variety of complex physiological processes, where they execute important functions, acting as cellular membrane components, signaling mediators, energy reserve molecules, and endocrine regulators [2–4]. The so-called lipid mediators are a group of regulatory molecules produced in response to extracellular stimuli that bind to their cognate receptors and transmit signals to target cells; their deregulation has been linked to various pathological conditions such as inflammation, atherosclerosis, and diabetes [5–7]. Lipid mediators include polyunsaturated fatty acids (PUFAs), lysophosphatidic acid, and their derivatives. It is believed that many regulatory activities of PUFAs are mediated by their oxidation products, oxylipins [8], which are produced and activated by several enzymes such as cytochrome P450 (P450), cyclooxygenases (COX), and lipoxygenases (LOX). Oxylipins have been mostly associated with the initiation and resolution of inflammation and vascular function. The kidneys have the highest levels of P450, COX, and LOX enzymes after the liver; these enzymatic pathways play a pivotal role in kidney function and renal disease [9–11]. The kidneys are essential to the urinary system and also play a role in systemic homeostasis. As urine production is regulated by oxylipins produced by the three major oxygenases, which respond to systemic conditions, the urinary oxylipin profile may reflect renal enzymatic activity as well as systemic status [12].

As an important and easily accessible biological fluid, urine has been the subject of detailed chemical analyses for more than 100 years. Even today, urine analysis is routinely performed using dipstick tests that can readily measure urinary glucose, bilirubin, ketone bodies, nitrates, leukocyte esterase, specific gravity, hemoglobin, urobilinogen, and protein. More detailed urinalysis can be also used to study a variety of renal conditions, including bladder and kidney cancer [13, 14]. However, some problems remain to be solved in urinary lipid-based diagnostics. Until recently, gas chromatography coupled with MS was the primary lipid analysis used for low-molecular-weight and low-polarity molecules, which usually require chemical derivatization. Several disease- and/or drug- related metabolic abnormalities can be detected in urine; however, little information is available on the urinary lipid composition in healthy subjects on a regular diet, and the normal variation in urinary lipid content has not been determined, which restricts the diagnostic potential of lipid profiling in urine samples despite its clinical significance.

To overcome these obstacles, we have attempted to characterize the human urine metabolome in healthy subjects. In this study, we performed cross-classification of urinary lipids by sex, age, and body mass index (BMI) in a healthy population after normal overnight fasting using liquid chromatography coupled with MS (LC/MS).

## Materials and Methods

### Collection and preparation of human urine

Urine samples from healthy white adults were purchased from PromedDX (Norton, MA). The samples were collected after written informed consent was obtained from all subjects. These were collected using the blood donor history questionnaires prepared by the AABB to screen for disease risk factors [15]. Therefore, all of these subjects are considered as Normal Healthy donors outlined by AABB. The ethics committee of the National Institute of Health Sciences authorized PromedDX as a validated provider of urine samples and exempted us from the committee’s approval for use of the purchased urine samples. All subjects declared healthy at the recruitment, and their urine was collected the next morning after fasting for 14 h from 60 individuals divided into four groups (n = 15): young men (25–33 years old), elderly men (55–64 years old), young women (25–34 years old), and elderly women (55–63 years old) (Table 1 and S1 Table). Fresh urine from each individual was collected into 15-ml test tubes, immediately frozen, and stored at -80°C. The samples were shipped from PromedDX on dry ice, thawed on ice after arrival, divided into small aliquots, and stored at -80°C until lipid extraction. Together with urine, fresh blood was simultaneously collected from same individuals, and the metabolites profiles in their plasma and serum are already published in our previous paper [16, 17]. There are no outliers in their serum creatinine levels [17], a classical biomarker for kidney function, suggesting their renal functions are normal.

**Table 1:** Demographic characteristics of the study population.

GROUP (number)	AGE±Std	BMI±Std
YOUNG MEN (n = 15)	29.0±2.4	26.2±5.6
ELDERLY MEN (n = 15)	59.0±2.57	24.5±4.7
MEN (n = 30)	44.0±15.1	25.1±5.2
YOUNG WOMEN (n = 15)	28.0±3.3	35.4±8.8
ELDERLY WOMEN (n = 15)	59.0±2.3	32.7±4.5
WOMEN (n = 30)	44.5±15.6	33.5±7.3
HIGH (>30) BMI male (n = 7)	32.0±16.2	33.1±1.9
HIGH (>30) BMI female (n = 21)	34.0±15.1	36.1±6.1
HIGH (>30) BMI (n = 28)	55.0±15.15	34.0±15.4
LOW (<30) BMI male (n = 23)	55.0±14.7	23.0±3.2
LOW (<30) BMI female (n = 9)	57.5±16.0	27.5±1.4
LOW (<30) BMI (n = 32)	35.36±5.73	25.3±3.3
TOTAL (n = 60)	44.5±15.3	29.2±7.7

Urine samples were collected from 60 individuals divided in four groups: young men, elderly men, young women, and elderly women (n = 15 per group), and also stratified by BMI, high (> 30) and low (< 30). The data on age and BMI are presented as the median ±STD. BMI, body mass index.

### Chemicals

Unless otherwise indicated, all reagents were of MS analytical grade. LC/MS grade methanol, acetonitrile, water, and chloroform were purchased from Wako Pure Chemical, Ltd. (Nihonbashi, Tokyo). 1,2-dipalmitoyl d6-3-sn glycerophosphatidylcholine (16:0/16:0 PC-d6) and ^13^C-labeled tripalmitin (tripalmitin-1,1,1-^13^C_3_) were purchased from Larodan Malmo (Solna, Sweden). Phospholipid, PUFA, and other oxylipin internal standards (ISs) were purchased from Cayman (Ann Arbor, MI).

### Sample preparation for MS analysis

Urine (200 μl) was added to 1,300 μl of methanol containing ISs used for normalization and evaluation of extraction efficacy. The following ISs were utilized: 16:0/16:0 PC-d6 (33.3 μM), tripalmitin-1,1,1-^13^C_3_ (3.3 μM), deuterated PGE_2_-d4 (33 pg/μl), and deuterated LTB_4_-d4 (33 pg/μl). The samples were centrifuged at 15,000 rpm for 3 min at 4°C to precipitate insoluble matter, and 1,440 μl of the supernatant was diluted 9.9-fold using Milli-Q water adjusted to pH 3.0 with 1N HCl and subjected to solid-phase extraction using Sep-Pak Vac. RC (500-mg) C18 cartridges (Waters, Milford, MA). The samples were then eluted sequentially with methyl formate and methanol. Lipid metabolites in the methanol fractions were measured by ultra-performance liquid chromatography/time-of-flight mass spectrometry (UPLC-TOFMS; LCT Premier XE; Waters Micro-mass; Waters) for analysis of phospholipids, sphingolipids, and neutral lipids [16]. PUFAs and their oxidation products (oxylipins) in the methyl formate fraction were measured by UPLC/MS/MS using a 5500QTRAP quadrupole-linear ion trap hybrid mass spectrometer (AB Sciex, Framingham, MA) interfaced with an ACQUITY UPLC System (Waters). Transitions were determined for all individual PUFAs and oxylipins using MRM mode (further information in S2 Table). Because tetranor-prostaglandin D metabolite (tPGDM) and tetranor-prostaglandin E metabolite (tPGEM) are constitutional isomers with indistinguishable precursor and product ion mass and the same retention time (S2 Table), it was not possible to distinguish them using our LC/MS/MS method. The recovery of analytes after extraction is summarized in S3 Table.

### Calibration curve

The external calibration curves were constructed by spiking the sample buffer (n = 3) with ten different concentrations (0.01 or 0.04–400 or 1000 nM) of analytes. Analyte concentrations are calculated as X (nM) to substitute [(IS normalized analyte area)/(extraction recovery)] for Y in polynomial expressions. The calibration data, including the polynomial expression of the approximate curve, calibration range, and correlation coefficient are all summarized in S3 Table.

### Data processing

LC-TOFMS data were processed using the 2DICAL software package (Mitsui Knowledge Industry Co. Ltd., Tokyo, Japan) as described by Ono et al. [18]. Extracted ion peaks were subjected to identification of lipid molecules by comparing ion features, including RT, m/z, preferred adducts, and in-source fragments, in the experimental samples with those in the reference library of lipid molecule entries, as described previously [16]. The peak height of the detected lipid molecules was corrected to that of deuterated PC as an IS in each sample. The corrected value for each lipid was then normalized to the corresponding creatinine levels in each urine sample (S4 and S5 Tables).

UPLC/MS/MS data on PUFAs and oxylipins were processed using MultiQuant^™^ Software (Version 2.1; AB Sciex). The integrated peak area of the detected lipid molecules was corrected to that of deuterated LTB_4_-d4 as an IS in each sample. The concentration in the correction-applied samples was determined by using the calibration curve and by adjusting the extraction efficiency as described above. The concentration of each lipid was then normalized to the corresponding creatinine levels in each urine sample and expressed as μmol analyte /mmol creatinine (S6 and S7 Tables) as described in the Human Metabolome Database (http://www.hmdb.ca/).

### ELISA

For urine volume normalization, the creatinine level in the urine of each subject was evaluated by enzyme-linked immunosorbent assay (TransGenic Inc., Kobe, Japan). Urine (10 μl) was diluted with 190 μl reaction buffer. The diluted sample was centrifuged at 1,500 rpm, and 70μl of the supernatant was transferred to a 96 well plate. After addition of 70 μl of reaction solution, the plate was incubated at RT for 1 h. After washing of the plate, chromogenic substrate solution (100 μl) was added to each well and incubated for 10 min. The absorption at 490 nm was analyzed using a microplate reader. Each plate comprised a calibration set and solvent blanks. (S5 and S7 Tables)

### Statistical analysis

Statistical analyses were performed in the R statistical environment (http://r-project.org/). Metabolite levels among the four groups were compared by the Mann-Whitney test, and differences were considered statistically significant at *p*<0.05. To adjust for multiple comparisons, the candidate compounds were narrowed down based on a false-discovery-rate (FDR) adjusted *q*<0.05.

## Results

### Lipid profiles in urine from healthy individuals

Exact mass measurement using electrospray ionization mass spectrometry (ESI-TOFMS) in the simultaneous negative and positive mode resulted in the identification of different phospholipids in the methanol fractions from human urine. There were at least 220 negative and 180 positive detectable ion peaks in human urine; however, only 14 phospholipids were identified. Among them, relative quantification was performed for two phosphatidylcholines (PCs), one sphingomyelin (SM), one phosphatidylinositol (PI), and three ceramides (Cer)s (S4 Table). Similar to most studies on urinary metabolites using LC/MS [19, 20], we did not detected phosphatidylethanolamine, phosphatidylserine, and neutral lipids. The strongest signals belonged to SM (d18:1/16:0), PC (16:0/18:2), and PC (16:0/18:1) (S1 Fig), and the levels of these molecules did not significantly vary according to sex, age, or BMI (S9 Table).

To quantify the lipids in the methyl formate fraction, the triple quadrupole LC/MS system was operated under the multiple reaction monitoring (MRM) mode using electrospray ionization in the negative ion mode. Of the 46 metabolites scanned in this target lipidomic analysis (S2 Table), four PUFAs and 25 oxylipins were observed at detectable and quantifiable levels in human urine. Similar to other studies [21], 9-hydroxyoctadecadienoic acid (HODE), 9-oxooctadecadienoic acid (KODE), 12- hydroxyeicosatetraenoic acid (HETE), docosahexaenoic acid (DHA), arachidonic acid (AA), and prostaglandin (PG) derivatives were detected at relatively high levels among the monitored ions (S6 Table).

### Higher urinary content of 12-LOX-produced oxylipins in female than men

**Fig 1:**
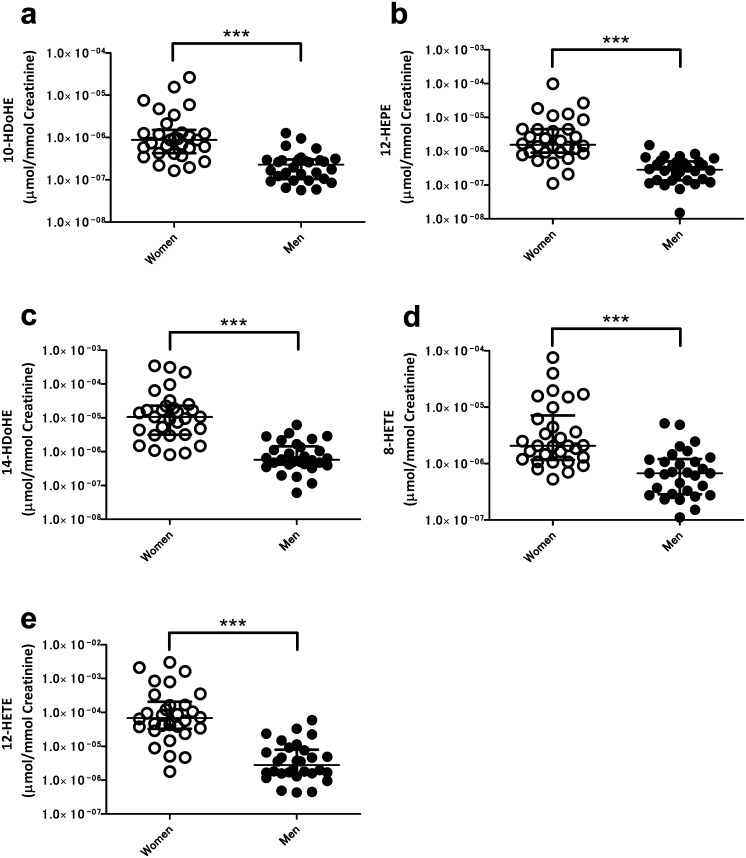
The association of urinary lipids with sex. Women had higher concentrations of 10-HDoHE (a), 12-HEPE (b), 14-HDoHE (c), 8-HETE (d) and 12-HETE (e) than men. Open circles and closed circles show women and men subjects, respectively. The center bars show median and interquartile values normalized to respective creatinine levels; ***p < 0.001, by the Mann-Whitney U-test.

To access impacts of physiological backgrounds on urinary lipid levels, we initially examined sex-related variations. Female urine contained high concentrations of three ω-3 oxylipins known to have anti-inflammatory and vasodilatory activities [22] [23] (Table 2 and S8 Table). Regardless of the double bond position, three 12-LOX-produced oxylipins were significantly enriched in female compared to male urine. The levels of 10-hydroxyldocosahexaenoic acid (HDoHE) (Fig 1a), 12- hydroxyeicosapentaenoic acid (HEPE) (Fig 1b), and 14-HDoHE (Fig 1c) were significantly higher in female urine. In agreement with previous reports [24], the levels of the urinary ω-6 fatty acid derivatives 8-HETE (Fig 1d) and 12-HETE (Fig 1e) were also significantly different between men and women.

**Table 2:** Sex-related oxylipins.

Compound	Δ	Precursor	Pathway	Tendency	Ratio	*P*	*Q*	Effect
10-HDoHE	ω3	DHA	-	Women>Men	3.8	***	^$ $ $^	Anti-inflammation/ vasodilation
12-HEPE	ω3	EPA	12-LOX	Women>Men	5.4	***	^$ $ $^	Anti-inflammation/ vasodilation
14-HDoHE	ω3	DHA	12-LOX	Women>Men	18.6	***	^$ $ $^	Anti-inflammation/ vasodilation
8-HETE	ω6	AA	P450	Women>Men	3.1	***	^$ $ $^	Inflammation/ vasodilation
12-HETE	ω6	AA	12-LOX	Women>Men	24.2	***	^$ $ $^	Inflammation/ vasodilation

Δ: The first double bond.

-: non-enzymatic.

Ratio represents the median ratio of the value in women to that in men.

***p < 0.001 by the Mann-Whitney U-test.

^$ $ $^*q*<0.001 by Benjamini-Hochberg procedure for FDR.

### Higher urinary anti-hypertensive oxylipin levels in women than in men of young age

**Fig 2:**
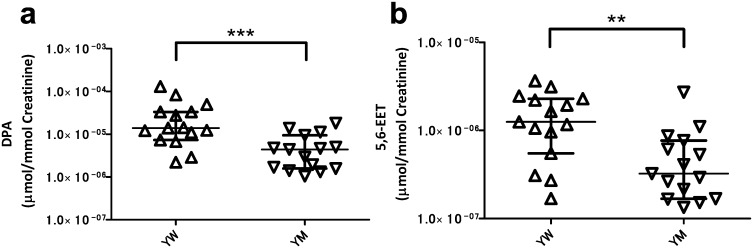
The gender-related urinary oxylipins in young age. Young women had higher concentration of DPA and 5,6-EET than young men (a and b). Open triangles and open inverted triangles show young women (YW) and young men (YM), respectively. The center bars show median and interquartile values normalized to the respective creatinine level. **p < 0.01, ***p < 0.001 by the Mann-Whitney U-test.

We identified a difference in the levels of several urinary oxylipins specific to young women (Table 3 and S8 Table). Young women showed high concentrations of five compounds with anti-hypertensive activities in their urine. Thus, compared to young men, young women had approximately 3–4 fold higher content of three PUFAs including AA, Docosapentaenoic acid (DPA) (Fig 2a), and Eicosapentaenoic acid (EPA). Three epoxyeicosatrienoic acids (EETs), which are P450 metabolites of AA [11], and included 11,12-EET, 14,15-EET, and 5,6-EET (Fig 2b) were also present in higher level in young women. DPA, EPA, and those EETs are anti-hypertensive mediators [25]. 15-HETE, a major AA metabolite [26], was also present at higher levels in the urine of young women.

**Table 3:** Young women- related oxylipins.

Compound	Δ	Precursor	Pathway	Tendency	Ratio	*P*	*Q*	Effect
DPA	ω3	ALA	PUFA	YW>YM	3.2	***	^$ $^	Anti-hypertensive
EPA	ω3	ALA	PUFA	YW>YM	3.5	**	^$^	Anti-hypertensive
AA	ω6	-	PUFA	YW>YM	5.1	*	^$^	
11,12-EET	ω6	AA	P450	YW>YM	2.4	*	^$^	Anti-hypertensive
14-15-EET	ω6	AA	P450/-	YW>YM	3.0	**	^$^	Anti-hypertensive
5,6-EET	ω6	AA	P450	YW>YM	3.9	**	^$^	Anti-hypertensive
15-HETE	ω6	AA	-	YW>YM	2.9	**	^$^	inflammation

Δ: The first double bond.

-: non-enzymatic.

YW: young women.

YM: young men.

The ratio represents the median ratio the values in young women to those in young men. ALA: linokenic acid.

*p < 0.05,

**p < 0.01,

***p < 0.001 by the Mann-Whitney U-test.

^$^*q*<0.05,

^$ $^*q*<0.01 by Benjamini-Hochberg procedure for FDR.

### Higher urinary anti-inflammatory 5-LOX-pathway oxylipin levels in women than in men of elderly age

**Fig 3:**
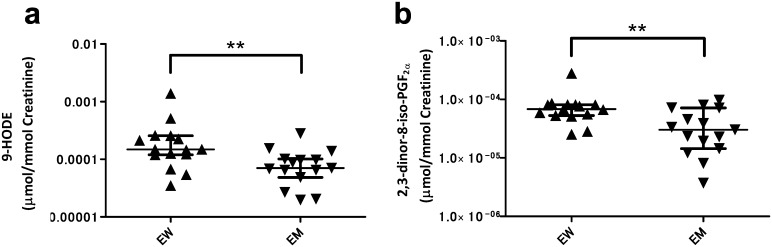
The gender-related urinary oxylipins in elderly age. Elderly women had higher concentration of 9-HODE and 2,3-dinor-8-iso-PGF_2α_ than other groups (a and b). Closed triangles, and closed inverted triangles show elderly women (EW) and elderly men (EM), respectively. The center bars represent median area values normalized to respective creatinine level. **p < 0.01 by the Mann-Whitney U-test.

We next assessed the association of urinary lipid levels with elderly women (Table 4). Three metabolites, 9-HOTrE (hydroxyoctadecatrienoic acid), 9-KODE (oxooctadecadienoic acid,) and 9-HODE (hydroxyoctadecadienoic acid) (Fig 3a), which belong to 5-LOX pathway oxylipins [27] and putative oxidative stress markers, showed approximately 2-fold higher levels in elderly women than in elderly men. The level of 2,3-dinor-8-iso-PGF_2α_, a non-enzymatic AA metabolite and an oxidative stress marker [28], was even greater in elderly women, too (Fig 3b).

**Table 4:** Elderly women-related oxylipins.

Compound	Δ	Precursor	Pathway	Tendency	Ratio	*P*	*Q*	Effect
9-HOTrE	ω3	ALA	5-LOX	EW>EM	2.2	*	^$^	Unknown
				EW>YW	0.3	**	^$^	
9-HODE	ω6	LA	5-LOX	EW>EM	2.1	**	^$^	Oxidative stress marker
9-KODE	ω6	LA	5-LOX	EW>EM	2.7	**	^$^	Oxidative stress marker
2,3-d-8-i-PGF_2α_	ω6	AA	-	EW>EM	2.2	**	^$^	Oxidative stress marker

2,3-d-8-i-PGF_2α_ 2,3-dinor-8-iso- PGF_2α_.

Δ: the first double bond.

-: non-enzymatic.

EW: elderly women.

EM: elderly men.

Ratio represents the median ratio of elderly women to elderly men.

ALA: linolenic acid.

*p< 0.05,

**p< 0.01 by the Mann-Whitney U-test.

^$^*q*<0.05 by Benjamini-Hochberg procedure for FDR.

### No difference in urinary oxypilin levels between ages and BMIs

Urinaly oxylipin levels were compared between the young and elderly subjects of both sex. As shown in S8 Table, there were no oxypilins showing age-related difference within women and men groups. We also assessed the association of BMI and with urinary lipid concentration. Comparing subjects with lower BMI (< 30) and those with higher BMI (> 30), none of the oxypilins showed significant association with BMI (S8 Table).

## Discussion

Our primary objective was to assess differences in urinary lipid levels between groups of healthy subjects classified by demographic variables of sex, age, and BMI to provide reference ranges for urinary lipidomics. Although there have been several reports on the association of urinary lipids with dietary control, disease status, and/or drug administration [29], this is the first report on cross-classification of urinary lipid content by age, sex, and BMI in healthy individuals after physiological fasting. Our results demonstrate that sex significantly impacted the measurement of urinary lipid metabolite levels in the healthy population. We previously performed serum and plasma lipid profiling in the same population investigated in the present study [16]; however, sex-related differences in the urinary oxylipins did not show a correspondence with those in serum/plasma oxylipins that we previously reported [16, 17]. In the present study, none of the analytes exhibit significant differences according to age or BMI, but 16 analytes showed sex differences in urine. Therefore, in measurement of urinary lipids, attention should be paid to the sex differences in at least these 16 analytes.

Oxylipins might be produced by the kidney because 12-HETE in the blood stream was not directly secreted into the urine in a previous study [30]. In the present study, oxylipins generated from ω-3 fatty acids had notably higher levels in female urine, which may be explained by the previous finding that the ω-3 conversion efficiency from linolenic acid (ALA) to longer-chain PUFAs is greater in women than in men [31]. Accordingly, anti-inflammatory and vasodilatory oxylipins had significantly higher concentrations in female urine than in male urine. 10-HdoHE, 12-HEPE, and 14-HDoHE are examples of potentially anti-inflammatory ω-3 fatty acids [32, 33]. Although the mechanisms underlying the observed sex differences remain unclear, our results suggest that 12-LOX is more active in women, accounting for the sex difference in oxylipin levels.

A recent study in spontaneously hypertensive rats revealed stronger renal vasodilator effects of AA, which could be accounted for by increased renal vascular sensitivity to EETs and K+ channel activation, leading to hyperpolarization and relaxation [25]. EETs are closely involved in the production of progesterone [34, 35], which may explain the difference observed between young and elderly women. There are no data on activation of EET synthesis by estrogen; however, dihydrotestosterone was previously shown to decrease EET levels [36], providing a possible reason why young women had higher urinary concentrations of EETs. Three oxylipins abundant in elderly women are regulated by the 5-LOX pathway [37]. 2,3-dinor-8-iso-PGF_2α_ is more abundant in human urine than its precursor, 8-iso- PGF_2α_ [38]. These oxidative stress markers might be particularly abundant in the urine of elderly women because of their comparatively higher BMI.

The unique oxylipin profile observed in urine can be attributed to the specific function that the kidneys perform by concentrating certain metabolites from blood. As the differential activity of renal enzymes is likely to be responsible for the differences in urinary oxylipins observed between the groups, urinary lipidome profiling could be an attractive approach for monitoring renal conditions and/or functional performance, especially considering the relative stability of urinary oxylipins [39]. These benefits could be used to enhance diagnostic confidence, precision, and reliability. The findings of this study, i.e, that urinary lipid metabolite levels in the healthy population vary across sexes, have implications for understanding the biochemical changes in urine associated with renal physiology and would contribute to the advancement of urine-based diagnostic strategies.

## Supporting Information

S1 Fig Analysis of urinary phospholipids by UPLC-LCT. (TIFF)

S1 Table Information on healthy urine donors. (XLSX)

S2 Table The list of MRM transitions (M1, precursor; M2, fragment; RT, retention times) for the analysis. (XLSX)

S3 Table Calibration to determine the analyte concentration in urine and extraction recovery. (XLSX)

S4 Table Main phospholipid levels in urine of healthy adults. (XLSX)

S5 Table Individual dataset of the normalized peak height of Identified phospholipids in human urine. (XLSX)

S6 Table Summarized oxylipin concentrations (μmol/mmol creatine) in urne of healthy adults. (XLSX)

S7 Table Individual dataset of oxylipin concentrations (μmol/mmol creatine) in urne of healthy adults. (XLSX)

S8 Table Sex, age, and BMI-related differences (fold changes and statistical analysis) in the levels of urinary oxlipins. (XLSX)

S9 Table Sex, age, and BMI-related differences (fold changes and statistical analysis) in the levels of lipid metabolites. (XLSX)
