# Supplementary figures and images for: Cross-Classification of Human Urinary Lipidome by Sex, Age, and Body Mass Index
Source: PLoS One. 2016 Dec 14;11(12):e0168188. doi: 10.1371/journal.pone.0168188 (PMC5156423; doi:10.1371/journal.pone.0168188)

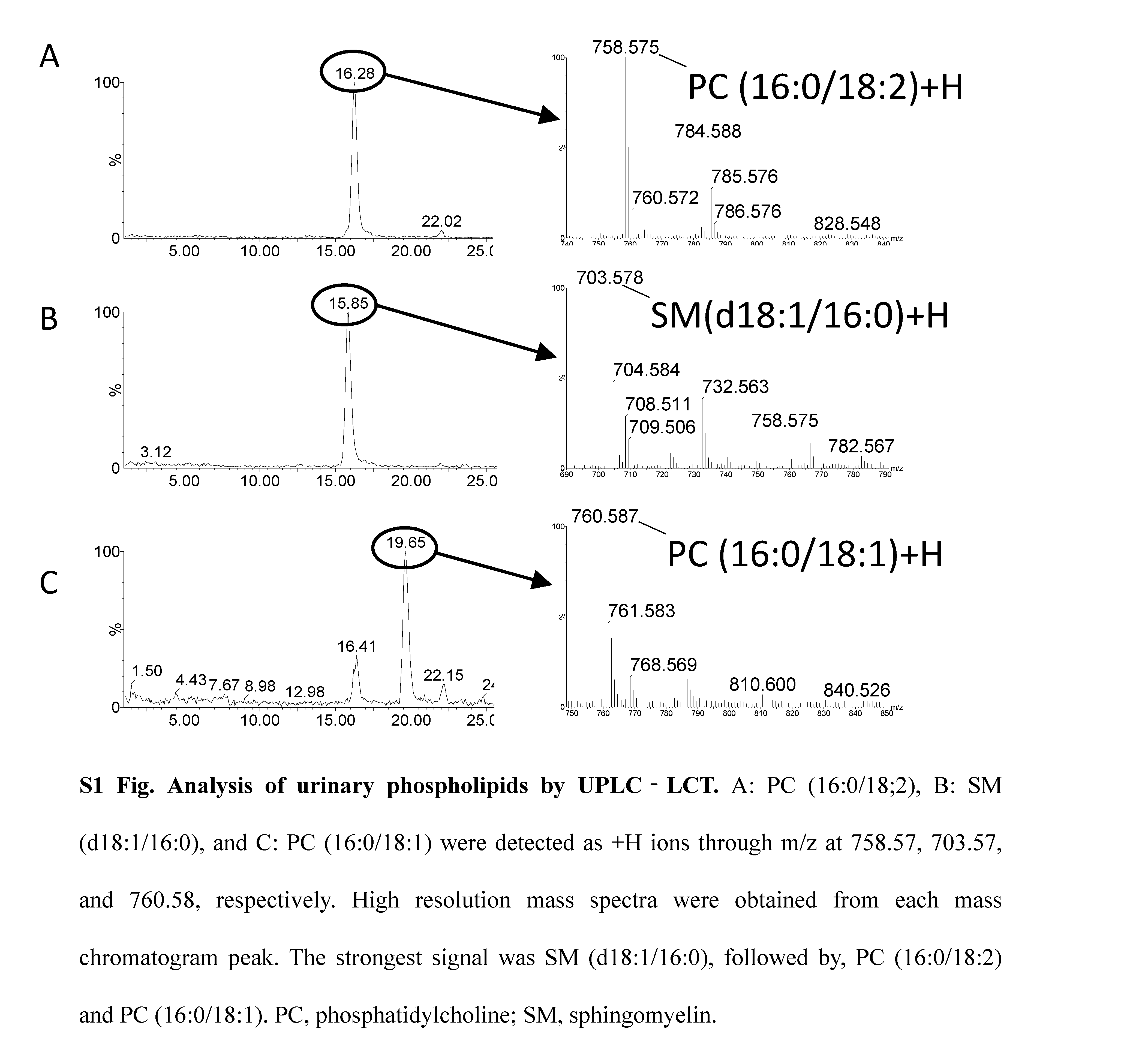

Supplement: S1 Fig — (TIFF) [file pone.0168188.s001.tiff]
